# A Phylogeny of the Family Poritidae (Cnidaria, Scleractinia) Based on Molecular and Morphological Analyses

**DOI:** 10.1371/journal.pone.0098406

**Published:** 2014-05-28

**Authors:** Yuko F. Kitano, Francesca Benzoni, Roberto Arrigoni, Yoshihisa Shirayama, Carden C. Wallace, Hironobu Fukami

**Affiliations:** 1 Seto Marine Biological Laboratory, Field Science and Education Center, Kyoto University, Shirahama, Wakayama, Japan; 2 Department of Marine Biology and Environmental Science, Faculty of Agriculture, University of Miyazaki, Miyazaki, Japan; 3 Deptartment of Biotechnology and Biosciences, University of Milano-Bicocca, Milan, Italy; 4 Japan Agency for Marine-Earth Science and Technology, Yokosuka, Kanagawa, Japan; 5 Museum of Tropical Queensland, Townsville, Queensland, Australia; Tel-Aviv University, Israel

## Abstract

The family Poritidae formerly included 6 genera: *Alveopora*, *Goniopora*, *Machadoporites*, *Porites*, *Poritipora*, and *Stylaraea*. Morphologically, the genera can be differentiated based on the number of tentacles, the number of septa and their arrangement, the length of the polyp column, and the diameter of the corallites. However, the phylogenetic relationships within and between the genera are unknown or contentious. On the one hand, *Alveopora* has been transferred to the Acroporidae recently because it was shown to be more closely related to this family than to the Poritidae by previous molecular studies. On the other hand, *Goniopora* is morphologically similar to 2 recently described genera, *Machadoporites* and *Poritipora*, particularly with regard to the number of septa (approximately 24), but they have not yet been investigated at the molecular level. In this study, we analyzed 93 samples from all 5 poritid genera and *Alveopora* using 2 genetic markers (the barcoding region of the mitochondrial COI and the ITS region of the nuclear rDNA) to investigate their phylogenetic relationships and to revise their taxonomy. The reconstructed molecular trees confirmed that *Alveopora* is genetically distant from all poritid genera but closely related to the family Acroporidae, whereas the other genera are genetically closely related. The molecular trees also revealed that *Machadoporites* and *Poritipora* were indistinguishable from *Goniopora*. However, *Goniopora stutchburyi* was genetically isolated from the other congeneric species and formed a sister group to *Goniopora* together with *Porites* and *Stylaraea*, thus suggesting that 24 septa could be an ancestral feature in the Poritidae. Based on these data, we move *G. stutchburyi* into a new genus, *Bernardpora* gen. nov., whereas *Machadoporites* and *Poritipora* are merged with *Goniopora*.

## Introduction

The family Poritidae Gray, 1842 is distributed throughout the tropics [1] and includes over 140 nominal species [2], some of which are among the major coral reef framework builders. The family formerly included 6 extant genera (currently *Alveopora* is not included), 3 of which are species-rich [1]—*Porites* Link, 1807 (73 species), *Goniopora* de Blainville, 1830 (31 spp.), and *Alveopora* de Blainville, 1830 (18 spp.)—and 3 are monospecific—*Stylaraea* Milne Edwards & Haime, 1851, *Poritipora* Veron, 2000, and *Machadoporites* Nemésio, 2005. These last 3 are rare or restricted to peculiar environmental conditions or have a limited geographic distribution. Conversely, the 3 major genera are common throughout the Indo-Pacific, with *Porites* extending into the Atlantic, from tropical to temperate regions. The biological, ecological and biometeorological studies such as climate change using *Porites* are well known [3]–[7], and this genus has been studied extensively at the molecular level in comparison with other coral genera [8]–[10] although it is still very difficult to divide *Porites* into discrete species in some cases (e.g., *P. lutea* and *P. lobata*)[9] due to the tiny but highly variable morphological characters used in the classification. In contrast, there are very few ecological and molecular studies of other Poritidae [11], [12], with the exception of our recent work showing the phylogenetic relationships of several species of *Goniopora* [13].

There has been some dispute over the classification of genera in the Poritidae since the revision by Dana [14]. In particular, the position of *Alveopora* has been contentious [15]. Vaughan and Wells [16] reinstated *Alveopora* in the Poritidae because of its similarity to *Goniopora* in ecology and polyp behavior, although in listing the skeletal characteristics of the genus, Wells [17] qualified most as “except in *Alveopora*”. *Alveopora* may be confused with *Goniopora* in the field because the polyps of both genera have very long columns, a character not shared with other genera, and these are usually elongated, obscuring the skeleton. As Veron and Pichon [15] noted, *Goniopora* polyps have 24 tentacles whereas *Alveopora* polyps have 12 tentacles. Moreover, they state that “no east Australian *Alveopora* shows any sign of having either of the patterns of septal fusion found in *Porites* or *Goniopora*.” In support of this argument, recent molecular studies have indicated that *Alveopora* is genetically distant from *Porites* and *Goniopora* [18], [19], although only a few specimens and species from each genus were analyzed in these studies. Based only on the evidence from these published genetic works, *Alveopora* was transferred from the Poritidae to the Acroporidae Verrill, 1902 by Dai and Horng [20], albeit without any formal diagnosis or discussion. Recently the characters of *Alveopora* have been discussed in relation to the Acroporidae [21], [22].

*Goniopora* is easily distinguishable from other genera in the Poritidae, with 3 septal cycles and 24 septa (vs. 2 septal cycles and 12 septa for *Porites*, *Alveopora*, and *Stylaraea*) and larger corallites [15]. *Machadoporites* has 15–22 septa and hence fewer than *Goniopora* [23]. *Poritipora* skeletal morphology is quite similar to that of *Goniopora* due to the presence of 24 septa, but the former has smaller corallites and only 2 septal cycles [24]. *Stylaraea* is the only monospecific poritid genus to have been studied since its description. One ecological study [25] and a few morphological studies [26] have been reported for *Stylaraea*. *Stylaraea punctata* is possibly the smallest colonial zooxanthellate scleractinian coral and has been reported as a brooder [25]. This genus superficially resembles *Porites*, but is distinguished from it by reduced septation without the *Porites* pattern of fusion (e.g. septal triplet), and the absence of pali, although its phylogenetic position in the Poritidae has not been studied.

Recent molecular phylogenetic analyses of scleractinian corals [19], [27]–[31] revealed that molecular-based phylogenetic relationships sometimes conflict with traditional macromorphology-based taxonomy. This indicates that the current, common identifying characters within a family or genus of scleractinian corals do not always reflect their phylogenetic relationships. One such inconsistency is seen in the relationship between Atlantic and Indo-Pacific Faviidae Gregory, 1900 [27] (but see [32] for the taxonomic revision). Despite such conflicts, there are also many cases in which molecular data are consistent with traditional taxonomy. Moreover, some morphological characters previously not considered key characters have been found to be effective as diagnostics for the phylogenetic relationships in several cases [32]–[34]. Overall, most molecular studies conclude that molecular analysis is useful in discerning the relationships of species or genera and inferring the phylogeny. Forsman et al. [9] investigated the molecular-based phylogenetic relationships among species of *Porites*, using mainly the entire internal transcribed spacer region (ITS) of the nuclear ribosomal DNA. Their results showed that most species of *Porites* were clearly distinguishable genetically, highlighting the usefulness of ITS markers in inferring relationships in *Porites* at the species level.

In this study, we assess the relationship of all 5 genera in the Poritidae with *Alveopora* to revise the taxonomy, and infer the morphological changes in the evolutional lineage in this family, using both molecular and morphological analysis. Also to assess phylogenetic variation in the regional and species differences, the present study examines a large number of specimens collected with broad geographic ranges from mainly Japan water to the Indian Ocean, covering most of common species and some uncommon and rare species, together with the genetic data of *Porites* spp. from Forsman et al. [9].

## Materials and Methods

### Collection

**Figure 1:**
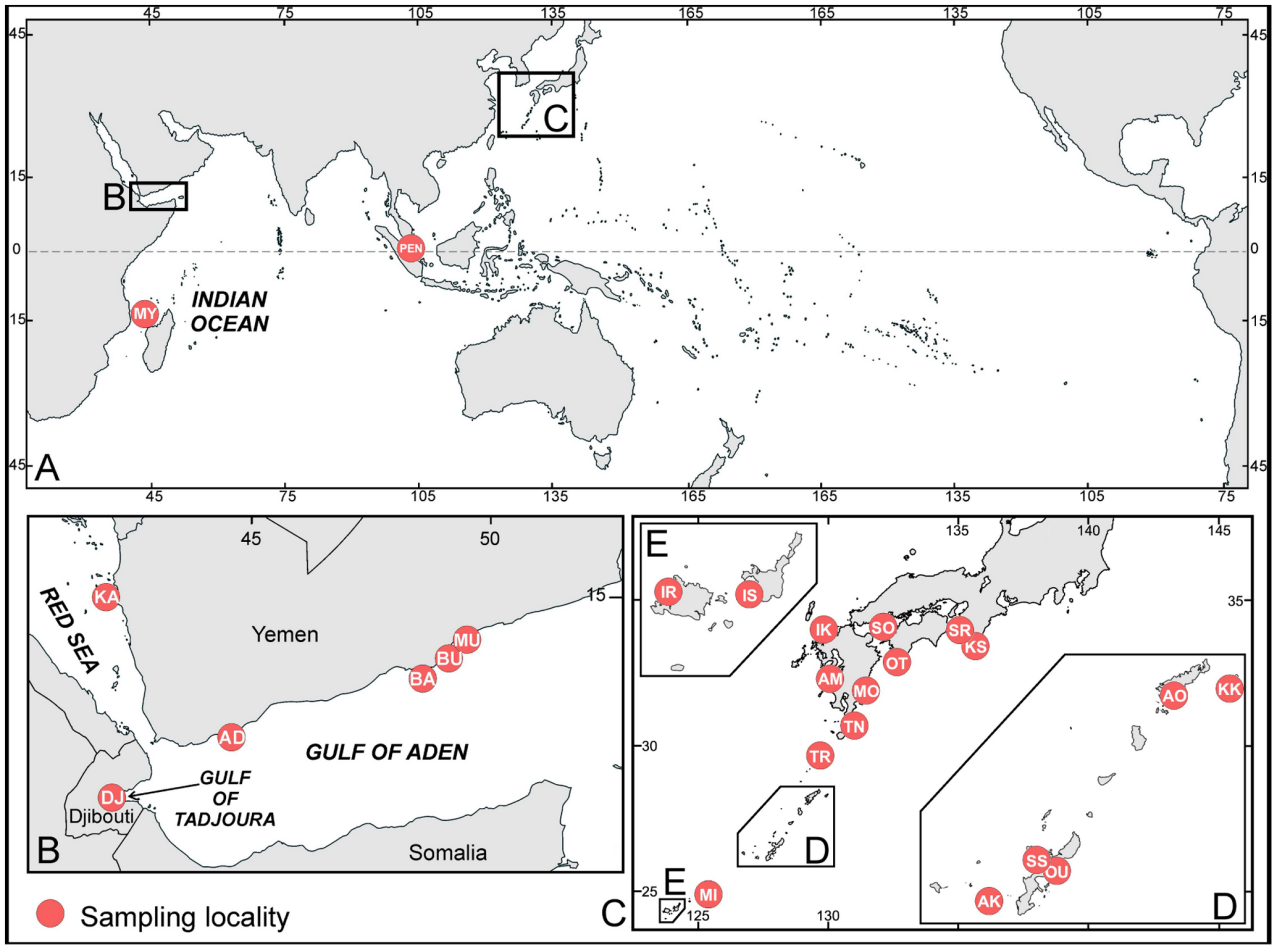
Map of sampling locations for this study. A: Indian and Pacific Ocean, B: southern Red Sea, Gulf of Tadjoura and Gulf of Aden; C–E: main island of Japan and Ryukyu archipelago. AD: Aden, Yemen, AK: Akajima Island, Japan, AM: Amakusa, Japan, AO: Amami-Oshima, Japan, BA: Bir Ali, Yemen, BU: Al Mukallah, Yemen, DJ: Djibouti, IK: Iki Island, Japan, IR: Iriomote Island or Hatoma Island, Japan, IS: Ishigaki Island or Taketomi Island, Japan, KA: Kamaran Islands, Yemen, KK: Kikai Island, Japan KS: Kushimoto, Japan, MI: Miyako Island, Japan, MY: Mayotte Island, France, OT: Ootuki, Japan, OU: Oura bay, Japan, PEN: Song song Island, Malaysia, SO: Suou-Oshima, Japan, SR: Shirahama, Japan, SS: Sesoko Island, Japan, TN: Tanegashima Island, Japan, TR: Nakanoshima Island, Japan

Most specimens (approximately 10 cm^3^ in size) were collected from 17 sites in Japan (Fig. 1). Additionally some samples were collected from Malaysia, and western Indian Ocean (Table 1). All Japanese sampling (AK, AM, AO, IK, IR, IS, KK, KS, MI, MO, OT, OU, SO, SR, SS, TN, TR; see Fig. 1) was performed in the frame of research projects by Japanese Society for Coral Taxonomy or by associate prof. H. Fukami at University of Miyazaki with sampling permission from each local government in Japan. Malaysia (PEN; see Fig. 1) sampling has taken place by local staffs in non-marine protected area, Songsong Island, under the permission of the research project by prof. Zulfigar Yasin and prof. Aileen Tan at Universiti Sains Malaysia. All western Indian Ocean sampling was also performed in the frame of research projects for which a sampling permission was delivered by local authorities and samples were shipped with CITES permits. AD, BA, BU, DJ, and MU are all sites in Yemen (Fig. 1). There, sampling has taken place in several missions and regular sampling permits were issued by Yemen Environmental Protection Agency (EPA) in Sana'a. Moreover, EPA staff supervised the activities in the field at all times. MY is Mayotte Island (Fig. 1). Sampling permits there were issued by the Direction de l'Agriculture et de la Foret de Mayotte, Service Environnement et Foret and by the Maritime Affairs Office. DJ are samples from Djibouti (Fig. 1) taken during the Tara Oceans expedition and the sampling permits were delivered by the Aménagement du Territoire et de l'Environnement de Djibouti. Photos of each specimen were taken in the field (particularly for living polyps) and the depth and habitat were recorded. After collection, a small piece of each specimen was removed for use in DNA extraction (see below), and the remaining sample was bleached to investigate the skeletal morphology for species identification.

**Table 1:** List of samples examined in this study and the accession numbers of DNA sequences.

Family	Species	DNA sample ID	specimen ID	locality	Country	COI (accession No.)	ITS (accession No.)
Acroporidae	*Montipora* sp.	SR127	MUFS YFK73	Shirahama	Japan	AB907075	-
Acroporidae	*Astreopora* sp.1	AO29	MUFS YFK444	Amami-Oshima	Japan	AB907076	-
Acroporidae	*Astreopora* sp.1	AO109	MUFS YFK485	Amami-Oshima	Japan	AB907077	-
Acroporidae	*Astreopora* sp.2	AO107	MUFS YFK484	Amami-Oshima	Japan	AB907078	-
Acroporidae	*Alveopora allingi*	AO125	MUFS YFK504	Amami-Oshima	Japan	AB907079	-
Acroporidae	*Alveopora allingi*	IR47	MUFS YFK1060	Iriomote	Japan	AB907080	-
Acroporidae	*Alveopora catalai*	AO117	MUFS YFK507	Amami-Oshima	Japan	AB907081	-
Acroporidae	*Alveopora catalai*	IR21	MUFS YFK971	Iriomote	Japan	AB907082	-
Acroporidae	*Alveopora excelsa*	KS5	MUFS YFK100	Kushimoto	Japan	AB907083	-
Acroporidae	*Alveopora excelsa*	OT26	MUFS YFK303	Otsuki	Japan	AB907084	-
Acroporidae	*Alveopora excelsa*	AM2	MUFS YFK365	Amakusa	Japan	AB907085	-
Acroporidae	*Alveopora japonica*	SO19	MUFS YFK1112	Suou-Oshima	Japan	AB907086	-
Acroporidae	*Alveopora japonica*	IK26	MUFS YFK226	Iki	Japan	AB907087	-
Acroporidae	*Alveopora japonica*	SR120	MUFS YFK66	Shirahama	Japan	AB907088	-
Acroporidae	*Alveopora japonica*	AM7	MUFS YFK366	Amakusa	Japan	AB907089	-
Acroporidae	*Alveopora* sp.	TN49	SMBL Cni10611	Tanegashima	Japan	AB907090	-
Acroporidae	*Alveopora* sp.	TN100	MUFS YFK1035	Tanegashima	Japan	AB907091	-
Acroporidae	*Alveopora spongiosa*	AO126	MUFS YFK510	Amami-Oshima	Japan	AB907092	-
Acroporidae	*Alveopora spongiosa*	SS15	MUFS YFK218	Sesoko	Japan	AB907093	-
Acroporidae	*Alveopora spongiosa*	IR145	MUFS HY11-40	Iriomote	Japan	AB907094	-
Acroporidae	*Alveopora tizardi*	SR50	MUFS YFK326	Shirahama	Japan	AB907095	-
Acroporidae	*Alveopora tizardi*	SR115	MUFS YFK58	Shirahama	Japan	AB907096	-
Acroporidae	*Alveopora verrilliana*	TR80	MUFS YFK689	Nakanoshima	Japan	AB907097	-
Acroporidae	*Alveopora verrilliana*	IS38	MUFS YFK943	Taketomi	Japan	AB907098	-
Dendrophylliidae	*Turbinaria peltata*	SR5	MUFS YFK126	Shirahama	Japan	AB907099	-
Dendrophylliidae	*Turbinaria peltata*	SR143	MUFS YFK89	Shirahama	Japan	AB907100	-
Dendrophylliidae	*Turbinaria peltata*	OT3	MUFS YFK279	Otsuki	Japan	AB907101	-
Poritidae	*Goniopora albiconus*	PEN1	MSL/USM/SS001	Songsong	Malaysia	-	AB906942
Poritidae	*Goniopora albiconus*	PEN9	MSL/USM/SS009	Songsong	Malaysia	-	AB906943
Poritidae	*Goniopora albiconus*	BA002	UNIMIB BA002	Bir Ali	Yemen	AB907026	AB906944
Poritidae	*Goniopora albiconus*	DJ074	UNIMIB DJ074	Oblal W	Djibouti	AB907027	AB906945
Poritidae	*Goniopora burgosi*	OT6	MUFS YFK286	Otsuki	Japan	AB907028	AB906946
Poritidae	*Goniopora* cf. *cellulosa*	TN29	MUFS YFK248	Tanegashima	Japan	AB907029	AB906947-51
Poritidae	*Goniopora* cf. *cellulosa*	TN54	SMBL Cni10573	Tanegashima	Japan	AB907030	AB906952
Poritidae	*Goniopora ciliatus*	BA076	UNIMIB BA076	Bir Ali	Yemen	-	AB906953
Poritidae	*Goniopora columna*	IS1	MUFS YFK351	Ishigaki	Japan	AB907031	AB906954
Poritidae	*Goniopora columna*	IS3	MUFS YFK353	Ishigaki	Japan	AB907032	AB748660*
Poritidae	*Goniopora djiboutiensis*	AK18	MUFS YFK163	Akajima Island	Japan	AB907033	AB906955-6
Poritidae	*Goniopora* cf. *fruticosa*	TN12	MUFS YFK263	Tanegashima	Japan	-	AB906957
Poritidae	*Goniopora* cf. *fruticosa*	TR1	MUFS YFK643	Nakanoshima	Japan	-	AB906958
Poritidae	*Goniopora* cf. *fruticosa*	TR4	MUFS YFK641	Nakanoshima	Japan	AB907034	AB906959
Poritidae	*Goniopora* cf. *fruticosa*	AO28	MUFS YFK443	Amami-Oshima	Japan	AB907035	AB906960
Poritidae	*Goniopora* cf. *fruticosa*	AO87	MUFS YFK458	Amami-Oshima	Japan	-	AB906961
Poritidae	*Goniopora lobata*	KK17	MUFS YFK813	Kikai	Japan	AB907036	AB906962
Poritidae	*Goniopora minor*	AO135	MUFS YFK513	Amami-Oshima	Japan	AB907037	AB906963
Poritidae	*Goniopora minor*	OU56	MUFS YFK773	Oura bay	Japan	AB907038	AB906964
Poritidae	*Goniopora minor*	MI6	MUFS KSMH20	Miyako	Japan	AB907039	AB906965
Poritidae	*Goniopora minor*	IR34	MUFS YFK988	Iriomote	Japan	-	AB906966
Poritidae	*Goniopora minor*	MY029	UNIMIB MY029	Ile Vert	Mayotte, France	-	AB906967
Poritidae	*Goniopora norfolkensis*	KK53	MUFS YFK843	Kikai	Japan	-	AB906968
Poritidae	*Goniopora norfolkensis*	AK2	MUFS YFK159	Akajima Island	Japan	AB907040	AB906969
Poritidae	*Goniopora norfolkensis*	IS7	MUFS YFK399	Ishigaki	Japan	AB907041	AB906970
Poritidae	*Goniopora* cf. *norfolkensis*	AK8	MUFS YFK165	Akajima Island	Japan	AB907042	AB906971
Poritidae	*Poritipora paliformis*	IS27	MUFS YFK940	Taketomi	Japan	AB907043	AB906972
Poritidae	*Poritipora paliformis*	IS48	MUFS YFK959	Taketomi	Japan	AB907044	AB906973
Poritidae	*Goniopora* cf. *pendulus*	PEN29	MSL/USM/SS029	Songsong	Malaysia	-	AB906974
Poritidae	*Goniopora somaliensis*	TR85	MUFS YFK690	Nakanoshima	Japan	AB907045	AB906975
Poritidae	*Goniopora somaliensis*	KK50	MUFS YFK841	Kikai	Japan	AB907046	AB906976
Poritidae	*Goniopora somaliensis*	OU29	MUFS YFK764	Oura bay	Japan	-	AB906977
Poritidae	*Goniopora somaliensis*	IR61	MUFS YFK1074	Hatoma	Japan	AB907047	AB906978
Poritidae	*Goniopora somaliensis*	DJ019	UNIMIB DJ019	Ras Ali	Djibouti	AB907048	AB906979-80
Poritidae	*Goniopora somaliensis*	DJ073	UNIMIB DJ073	Oblal W	Djibouti	-	AB906981
Poritidae	*Goniopora somaliensis*	DJ198	UNIMIB DJ198	Ankali Outer	Djibouti	-	AB906982-3
Poritidae	*Goniopora* cf. *somaliensis*	MY100	UNIMIB MY100	N'goudja inner barrier	Mayotte, France	AB907049	AB906984
Poritidae	*Goniopora stokesi*	BU034	UNIMIB BU034	Burum	Yemen	AB907050	AB906985
Poritidae	*Goniopora stokesi*	BU039	UNIMIB BU039	Burum	Yemen	-	AB906986
Poritidae	*Goniopora stokesi*	BU063	UNIMIB BU063	Burum	Yemen	-	AB906987
Poritidae	*Machadoporites tantillus*	AD068	UNIMIB AD068	Aden	Yemen	AB907051	AB906988
Poritidae	*Machadoporites tantillus*	BA032	UNIMIB BA032	Bir Ali	Yemen	AB907052	AB906989
Poritidae	*Machadoporites tantillus*	BA070	UNIMIB BA070	Bir Ali	Yemen	AB907053	AB906990
Poritidae	*Machadoporites tantillus*	BA099	UNIMIB BA099	Bir Ali	Yemen	AB907054	AB906991
Poritidae	*Goniopora tenuidens*	AM85	MUFS YFK1157	Amakusa	Japan	AB907055	AB906992
Poritidae	*Goniopora tenuidens*	KS15	MUFS YFK319	Kushimoto	Japan	AB907056	AB906993
Poritidae	*Goniopora tenuidens*	OT15	MUFS YFK297	Otsuki	Japan	AB907057	AB906994
Poritidae	*Goniopora tenuidens*	OT18	MUFS YFK298	Otsuki	Japan	AB907058	AB906995
Poritidae	*Goniopora tenuidens*	TN53	MUFS YFK50	Tanegashima	Japan	AB907059	AB906996
Poritidae	*Goniopora tenuidens*	KK28	MUFS YFK810	Kikai	Japan	-	AB906997
Poritidae	*Goniopora tenuidens*	IS18	MUFS YFK934	Taketomi	Japan	AB907060	AB906998
Poritidae	*Goniopora* sp.1	BA078	UNIMIB BA078	Bir Ali	Yemen	-	AB906999
Poritidae	*Goniopora* sp.2	MU139	UNIMIB MU139	Al Mukallah	Yemen	-	AB907000
Poritidae	*Goniopora stutchburyi*	KS1	MUFS YFK101	Kushimoto	Japan	AB907061	AB907001-3
Poritidae	*Goniopora stutchburyi*	KS50	MUFS YFK148	Kushimoto	Japan	AB907062	AB907004
Poritidae	*Goniopora stutchburyi*	MO33	MUFS YFK866	Miyazaki-Oshima	Japan	-	AB907005
Poritidae	*Goniopora stutchburyi*	MO99	MUFS YFK1184	Kushima	Japan	-	AB907006
Poritidae	*Goniopora stutchburyi*	OU5	MUFS YFK614	Oura bay	Japan	-	AB907007-10
Poritidae	*Goniopora stutchburyi*	OU40	MUFS YFK768	Oura bay	Japan	AB907063	AB907011
Poritidae	*Goniopora stutchburyi*	OU51	MUFS YFK771	Oura bay	Japan	-	AB907012
Poritidae	*Goniopora stutchburyi*	SS21G	MUFS YFK220	Sesoko	Japan	AB907064	AB907013
Poritidae	*Goniopora stutchburyi*	PEN2	MSL/USM/SS002	Songsong	Malaysia	AB907065	AB907014
Poritidae	*Goniopora stutchburyi*	PEN5	MSL/USM/SS005	Songsong	Malaysia	-	AB907015
Poritidae	*Goniopora stutchburyi*	PEN22	MSL/USM/SS022	Songsong	Malaysia	-	AB907016
Poritidae	*Goniopora stutchburyi*	SS21G	MUFS YFK220	Sesoko	Japan	AB907064	AB907013
Poritidae	*Stylaraea punctata*	AK88	MUFS YFK1239	Akajima	Japan	AB907066	AB907017
Poritidae	*Stylaraea punctata*	AK89	MUFS YFK1240	Akajima	Japan	AB907067	AB907018
Poritidae	*Stylaraea punctata*	AK90	MUFS YFK1241	Akajima	Japan	AB907068	AB907019
Poritidae	*Stylaraea punctata*	AK91	MUFS YFK1242	Akajima	Japan	AB907069	AB907020
Poritidae	*Stylaraea punctata*	AK92	MUFS YFK1243	Akajima	Japan	AB907070	AB907021
Poritidae	*Stylaraea punctata*	AK93	MUFS YFK1244	Akajima	Japan	AB907071	AB907022
Poritidae	*Stylaraea punctata*	AK101	MUFS YFK1245	Akajima	Japan	AB907072	AB907023
Poritidae	*Porites* cf. *lichen*	SR128	MUFS YFK74	Shirahama	Japan	AB907073	AB907024
Poritidae	*Porites* sp.	AK32	MUFS YFK194	Akajima	Japan	AB907074	AB907025

Dash means no data.

Asterisk shows accession number referred from Kitano et al. [13]. Note that more than one ITS sequences were obtained by sub-cloning from a single specimen in several samples while ITS from other samples were determined by direct sequencing of PCR products. Museum abbreviations are as follows: MSL/USM: Universiti Sains Malaysia, MUFS: University of Miyazaki, Department of Fisheries Science ( = Department of Marine Biology and Environmental Science), Japan, SMBL: Seto Marine Biological Laboratory, Kyoto University, Japan, and UNIMIB: University of Milano-Bicocca, Department of Biotechnology and Biosciences, Italy.

### Species identification

Species identification of *Goniopora* and *Alveopora* is difficult due to very limited skeletal characters and highly variable skeletal and polyp morphologies. The best solution to this common problem in the scleractinian corals is detailed analyses of the type material of each species [28], [29]. In order to minimize the risk of misidentification in this study, we firstly made lists of characters for species identification for these two genera (Tables S1, and S2) using the original descriptions and related references [1], [14], [15], [26], [35]–[60]. These tables were used to identify our specimens to species, examining the skeletal morphology of each specimen using a VHX-1000 digital microscope (Keyence) or stereoscopic microscope. Traditionally, species identification of *Goniopora* and *Alveopora* is based on skeletal characters, but recently Veron and Pichon [15], Nishihira and Veron [59], and Veron [1] added polyp characters to support species identification. Therefore, we also considered polyp characters along with skeletal characters for species identification in this study. The skeletal specimens collected in Japan are retained at University of Miyazaki (MUFS) or Seto Marine Biological Laboratory (SMBL). Skeletal specimens collected in Malaysia are deposited at Universiti Sains Malaysia (MSL/USM), and skeletal specimens collected in western Indian Ocean are at University of Milano-Bicocca (UNIMIB).

### Genetic analyses

A small sample (less than 1 cm^3^) of each specimen was put in CHAOS solution to dissolve the tissues or fixed in 99% ethanol. Total DNA was extracted from CHAOS solution using the phenol/chloroform extraction method [61], and from the coral tissues preserved in ethanol using the DNeasy Blood & Tissue Kit (Qiagen). The barcoding region of the mitochondrial Cytochrome oxidase subunit I (COI) was amplified by the polymerase chain reaction (PCR) using the primers ZCO1 and ZCO1R [9]. The nuclear ribosomal ITS region (ITS) including the 3′ end of the 18s rRNA, ITS-1, 5.8s, ITS-2, and the 5′ end of the 28s rRNA was also amplified by PCR using the primers 1S and 2SS [62]. The PCR condition for these two markers was 94°C for 30 seconds followed by 30 or 35 cycles at 94°C for 30 seconds, 55°C or 60°C for 45 seconds, and 72°C for 90 seconds, with a final phase of 72°C for 5 minutes. For the mitochondrial region, PCR products were treated with Shrimp Alkaline Phosphatase (SAP) and Exonuclease I (ExoI) at 37°C for 40 minutes followed by 80°C for 20 minutes. The DNA sequences were then determined via a direct sequence method, using ABI3730 or ABI310 sequencer. PCR products of the nuclear marker were also directly sequenced, but when obtained sequences had more than double peaks in the chromatogram, they were sub-cloned into TA-vector (Promega) or TOPO10 (Invitrogen) and sequenced using ABI3730 or ABI310. All DNA sequences obtained in this study were submitted to DDBJ (accession No. AB906942–AB907101, listed in Table 1).

A total of 15 COI and 26 ITS *Goniopora* sequences were taken from our previous study ([13], see Table S3). Also, a number of sequences were downloaded from GenBank/DDBJ and included in the molecular analyses. For COI analysis, 30 sequences from 18 species of *Porites* ([9], Table S3), one sequence (AB441211 [19]) from *Siderastrea siderea* in the family Siderastreidae Vaughan an Wells, 1943, two sequences (AB441201–AB441202 [19]) from *Galaxea fascicularis* in the family Euphylliidae (Alloiteau, 1952), and two sequences (AB441216–AB441217 [19]) from *Pavona* spp. in the family Agariciidae Gray, 1847 (species name of AB441216 is registered as *P. cactus* in DDBJ, but it is revised *P. decussata* as the results of the reexamination of the skeleton). Also, in the family Acroporidae, four sequences (AF338425, AY451340–AY451342 [63], [64]) from three species of *Acropora*, one sequence (AY903296 [65]) from *Montipora cactus* and one sequence (AY903295 [65]) from *Anacropora matthai* were used. For ITS, 67 sequences from 18 species of *Porites* from previous study ([9], see Table S3) were used. Because there are many sequences of *Porites* for COI and ITS registered in GenBank, the sequences of representative species from each of the different phylogenetic groups reported by Forsman et al. [9], were selected for this study. Finally, as Fukami et al. [19] and Kitahara et al. [31] showed that the family Dendrophylliidae Gray, 1847 is genetically closely related to the family Poritidae, specimens of *Turbinaria peltata* (family Dendrophylliidae) from Japan were also sampled for this study.

### Molecular phylogenetic analysis

Electropherograms and DNA sequences were checked and edited using Sequencher (Gene Code Co.) and SeeView 4.3.0 [66]. DNA sequences were aligned with MAFFT 7 [67] using the L-INS-i option. Then, all sites with indels and several sites with alignment ambiguities were excluded manually from the subsequent analyses. Two aligned DNA datasets (COI and ITS) used in this study are shown in supplementary information (Datasets S1, S2). Pairwise genetic distances were calculated as p-distance using MEGA 4.0.2 [68]. Phylogenetic trees were reconstructed by neighbor-joining (NJ) and maximum likelihood (ML). For NJ, PAUP* 4.0b10 [69] was used to infer the topologies for both COI and the ITS markers using Kimura 2-parameter model [70] and to conduct bootstrap analysis (1000 replicates). For ML, we assumed a model of nucleotide evolution obtained by using the Akaike Information Criterion (AIC) as implemented in MrModeltest 2.2 [71]. The most appropriate models of nucleotide evolution were TrN with invariant (I) and gamma (G) parameters (TrN+I+G) for the COI marker, and TrNef +I+G for ITS marker. PAUP* was used to reconstruct a best ML tree using a heuristic search and the tree-bisection-reconnection branch swapping method. GARLI (Genetic Algorithm for Rapid Likelihood Inference) 0.951 [72] was preferred to PAUP* for the bootstrap estimation as the former is less time consuming. Using GARLI, optimal ML topologies were searched with default setting using the models selected by MrModeltest (TrN+I+G for COI, TrNef +I+G for ITS) and bootstrap analyses (500 replicates) were conducted for each marker. MrBayes 3.2.2 [73] was also used to conduct Bayesian analyses under the same models. Four parallel chains of 1–4×10^6^ generations were run for each marker. Trees were sampled every 100 generations, and the initial 25% of the total trees as burn-in were discarded. The remaining trees were pooled to produce a 50% majority rule consensus tree. The average standard deviation of split frequencies after 4×10^6^ generations was 0.002069 for COI, and ones after 2.4×10^6^ generations was 0.009967 for ITS. All topologies obtained in these analyses were quite similar, so that only the ML tree inferred using PAUP* is used in this study.

In addition, we combined COI and ITS data and analyzed them with same methods as each marker using the GTR+I+G model for the nucleotide substitution (the average standard deviation of split frequencies after 1.0×10^6^ generations was 0.009909).

### Nomenclature Acts

The electronic edition of this article conforms to the requirements of the amended International Code of Zoological Nomenclature, and hence the new names contained herein are available under that Code from the electronic edition of this article. This published work and the nomenclatural acts it contains have been registered in ZooBank, the online registration system for the ICZN. The ZooBank LSIDs (Life Science Identifiers) can be resolved and the associated information viewed through any standard web browser by appending the LSID to the prefix “http://zoobank.org/”. The LSID for this publication is: urn:lsid:zoobank.org:pub: 6975D790-3A4F-466A-ABFA-D922E6675B4B. The electronic edition of this work was published in a journal with an ISSN, and has been archived and is available from the following digital repositories: PubMed Central, LOCKSS.

## Results

### Species identification

Twenty samples of *Alveopora* and 58 samples of *Goniopora* were analyzed in this study (Table 1). Although a few species have species-specific polyps, such as *Goniopora albiconus*, polyp characters vary greatly in the field. For example, terete tentacles, a typical polyp character of *G. tenuidens*, are also seen in *G. burgosi*.

**Figure 2:**
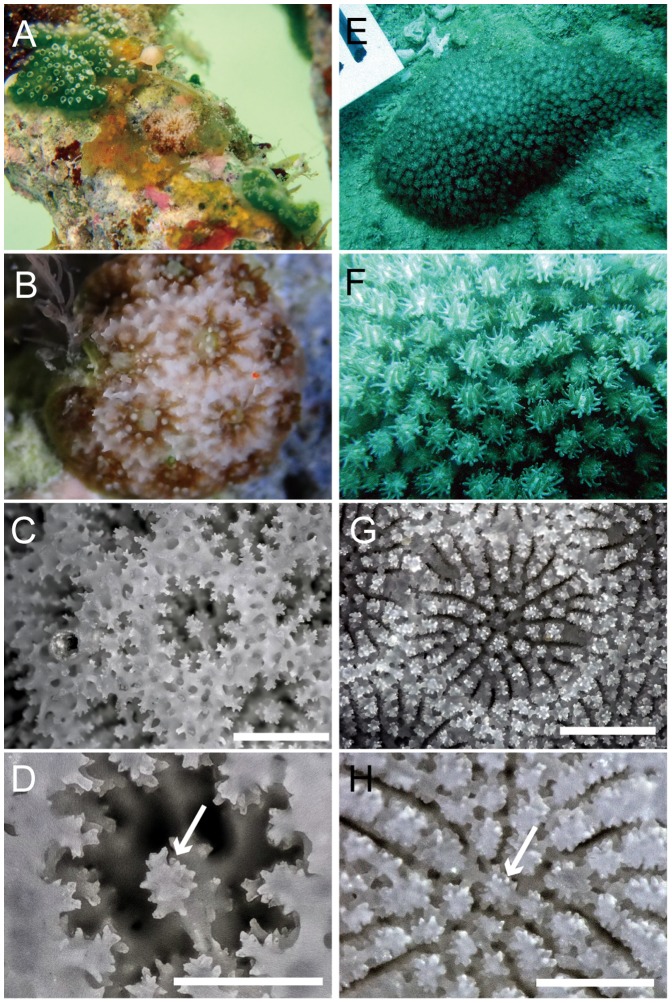
Polyp and skeleton characters of *Stylaraea punctata* and *Bernardpora stutchburyi*. A–B. *Stylaraea punctata* AK93, MUFS YFK1244, Akajima Island, Japan. C–D. *S. punctata* AK92, MUFS YFK1243, Akajima Island, Japan. E–H. *Bernardpora stutchburyi* SS21G MUFS YFK220, Sesoko, Japan. Living specimen for whole colonies (A, E) and polyps (B, F), corallite structures (C, G), and star-shaped columella (D, H). Arrows show columella. Bars show 1 mm for (C) and (G), and 0.5 mm for (D) and (H).

All 7 specimens of *Stylaraea punctata* were found in very shallow water (1 m) on a sandy beach in Aakajima Island, Okinawa, Japan (Fig. 1). Notably, all of them were attached to dead coral skeletons of the genus *Acropora*. Their size is very small (less 1 cm) and they have only 5 or 6 corallites. Tentacle and septal numbers were both 12 in all of them (Fig. 2A–D).

**Figure 3:**
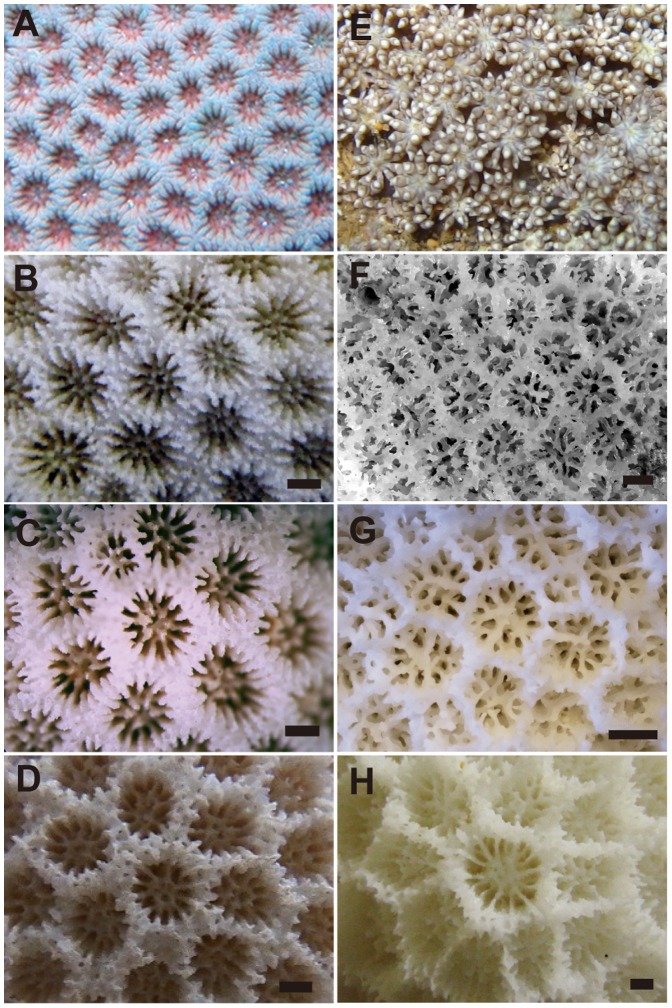
Polyp and skeleton characters of ‘*Poritipora*’ *paliformis*, ‘*Machadoporites*’ *tantillus* and morphologically related species. Living specimens and corallite structures for *P. paliformis* IS48, MUFS YFK959, Taketomi, Japan (A, B) and *M. tantillus* AD068, UNIMBI AD068, Aden, Yemen (E, F), respectively. Corallite structures of holotypes of *P. paliformis* MTQ G55857 (C) and *Goniopora minor* NHMUK 1934.5.14.436 No. 56 (D). Corallites structures of *G. burgosi* OT6, MUFS YFK286, Otsuki, Japan (G) and *G. pendulus* TN11, MUFS YFK243, Tanegashima, Japan (H) from Japan water, as examples of corallites with less 24 septa. Bars show 1 mm.

*Poritipora paliformis* Veron, 2000 has 24 septa with typically 2 septal cycles (long and short), 6 pali and no columella reported in the literature [24]. Two samples we collected in Taketomi Island, Japan (first record in the Pacific Ocean) had no elongating polyps in the field and had a cellular appearance (Fig. 3A), which is a feature of *P. paliformis*, as shown in Veron [1], [24]. The skeletal morphologies are also consistent in the literature, although the second cycle is not well developed in some corallites; however, many had 24 septa with 2 cycles (Fig. 3B). Therefore, we identified these 2 samples as *P. paliformis*. This species was described in Veron [1] without designating type material, and then it was redescribed [24] designating the holotype. However, the hototype of this species is not valid following ICZN [74], and the specimens listed in Veron [1] are regarded as part of the syntype series. Therefore, the holotype of this species listed in Veron [24] is to be considered a lectotype.

Four specimens collected in the Gulf of Aden (Fig. 3EF), which is near the type locality of *Machadoporites tantillus* (Claereboudt & Al-Amri, 2004), were identified as *M. tantillus* because they are consistent with the original description of this species [23].

Diagnostic morphological characters among genera in the family Poritidae are summarized in Table 2.

**Table 2:** Comparison of the diagnostic morphological characters between the previous classifications and the classification used in this study in the family Poritidae.

Genus	Calice diameter (mm)	Number of septa	Septal arrangement	Columella structure	References
**Previous study**					
*Porites*	0.5–2.2	12	Yes^1^	Central rod (tubercle) with a star-shaped granules if it develops	[1], [2], [15], [16], [78]
*Goniopora*	1.0–10.0	24	Yes^3^	Trabecular (fused septal dentations) if it develops	[1], [2], [15], [16], [26]
*Stylaraea*	<1	12	No	Central rod (tubercle) with a star-shaped granules	[1], [15], [46], [78]
*Poritipora*	2.0–2.5	24	No^2^	No	[1], [24]
*Machadoporites*	1.5	15–22	No	No	[81]
**This study**					
*Goniopora* *	1.0–10.0	12–24	Yes^3^/No^2^	trabecular (fused septal dentations) if it develops	This study
*Bernardpora* gen. nov.	<2	24	Yes^3^	Central rod (tubercle) with a star-shaped granules	This study

1 Specific septal pattern for *Porites*, ^2^3rd septa turn into 2nd septa ( = irregular reducing of gonioporoid pattern), ^3^Gonioporoid pattern.

*including *Poritipora* and *Machadoporites* as junior synonym.

### COI phylogeny

We obtained 69 COI sequences from all 5 genera in the Poritidae with *Alveopora*, 3 sequences from *Turbinaria peltata* and *Astreopora* spp., and one from *Montipora venosa* (Table 1). A total of 473 positions were used (120 polymorphic sites with 109 informative sites) and no indels were observed. A phylogenetic tree was reconstructed using these data, including sequences from GenBank/DDBJ (see Methods). *Siderastrea siderea* was used as an outgroup, based on the phylogenetic position of the Scleractinia shown by Fukami et al. [19].

**Figure 4:**
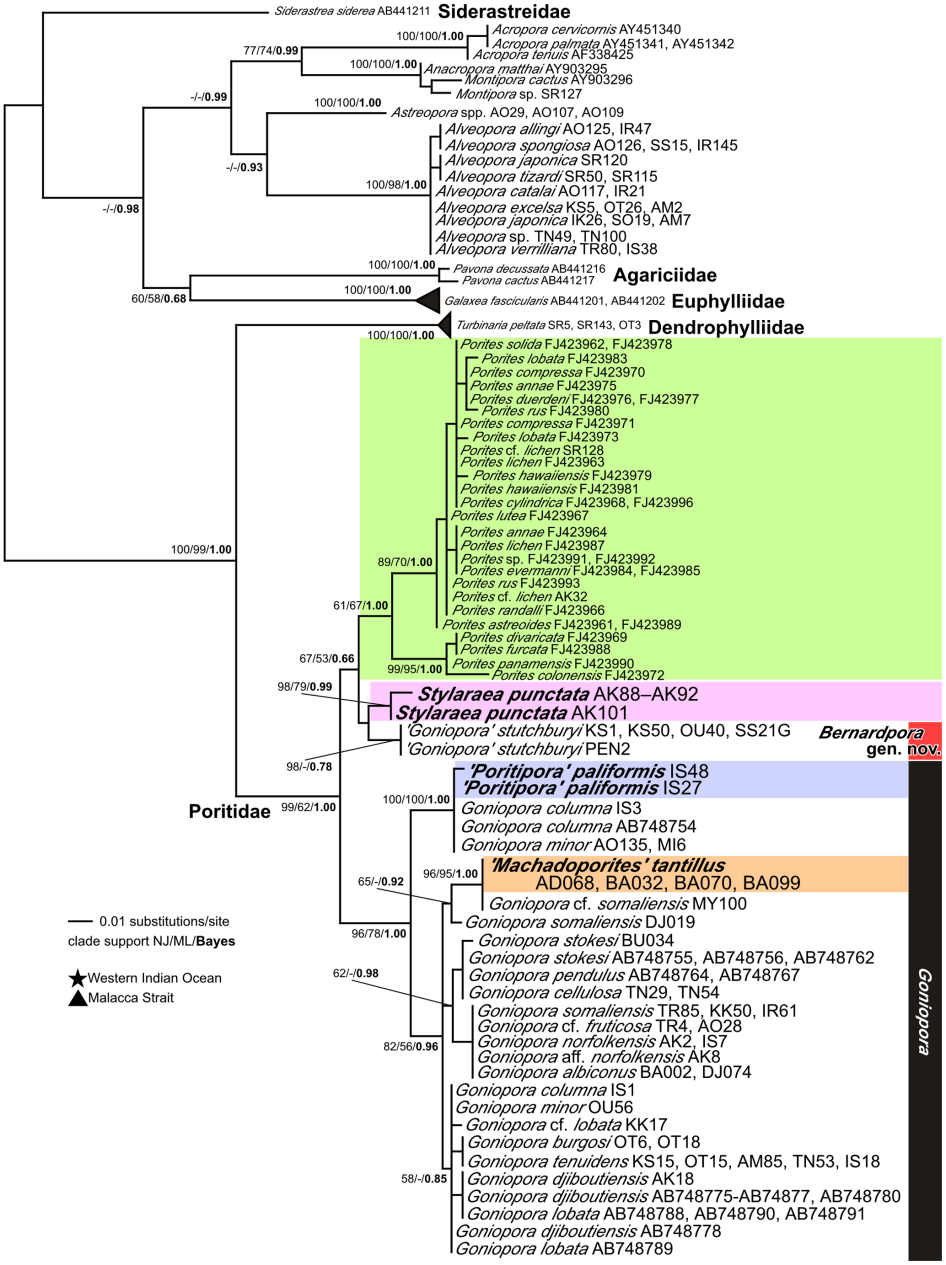
Molecular phylogenetic relationships of the family Poritidae and related families based on mitochondrial COI sequences. Numbers on/below main branches show bootstrap values (>50%) in ML and NJ analyses, and Bayesian posterior probability (>0.8). Stars show specimens collected from western Indian Ocean, and triangles show ones collected from Malacca Strait. Sample codes or accession numbers are shown after species names (see Table 1, Table S3). Grey in color for *Alveopora*, green for *Porites*, purple for *Stylaraea*, blue for ‘*Poritipora*’, and orange for ‘*Machadoporites*’. *Goniopora* is shown by bars in black. *Bernardpora* is shown by bar in red.

The COI phylogenetic tree showed that all 18 species of *Porites* are monophyletic. Moreover, the 13 *Goniopora* species we examined in this tree are also monophyletic, with the notable exception of *G. stutchburyi* (Fig. 4). This species and *Stylaraea punctata* are sister taxa (Fig. 2), and together they form a sister group to *Porites*. *Machadoporites tantillus* and *Poritipora paliformis* are nested within *Goniopora* (except *G. stutchburyi*). On the one hand, within the *Goniopora* phylogeny, *M. tantillus* forms a clade with the western Indian Ocean species *G. somaliensis* and *G.* cf. *somaliensis*. On the other hand, *P. paliformis* forms a clade with *G. minor* and *G. columna*, although *G. somaliensis* and *G. minor* are polyphyletic. Notably, all *G. somaliensis* from Japan are genetically distant from the *G. somaliensis* specimens from the western Indian Ocean. Uncorrected genetic p-distances between *G. stutchburyi* and *Porites* and between *G. stutchburyi* and the remaining *Goniopora* spp. were very similar (approximately 0.02).

All samples of *Alveopora* are genetically distant from all other poritids (p-distance 0.08–0.10), but closely related to the family Acroporidae (0.06). The phylogenetic position of *Alveopora* is unclear due to low bootstrap values, but it forms a sister group with *Astreopora* spp. In addition, sequences from *T. peltata* (family Dendrophylliidae) form a sister clade of all poritids except *Alveopora* and are positioned between *Alveopora* and the other poritids.

### ITS phylogeny

We obtained a total of 84 sequences of ITS from all 5 genera in the Poritidae (Table 1). In this study, we excluded *Alveopora* from ITS analysis because ITS regions were highly variable between *Alveopora* and other genera and they were hardly aligned.

**Figure 5:**
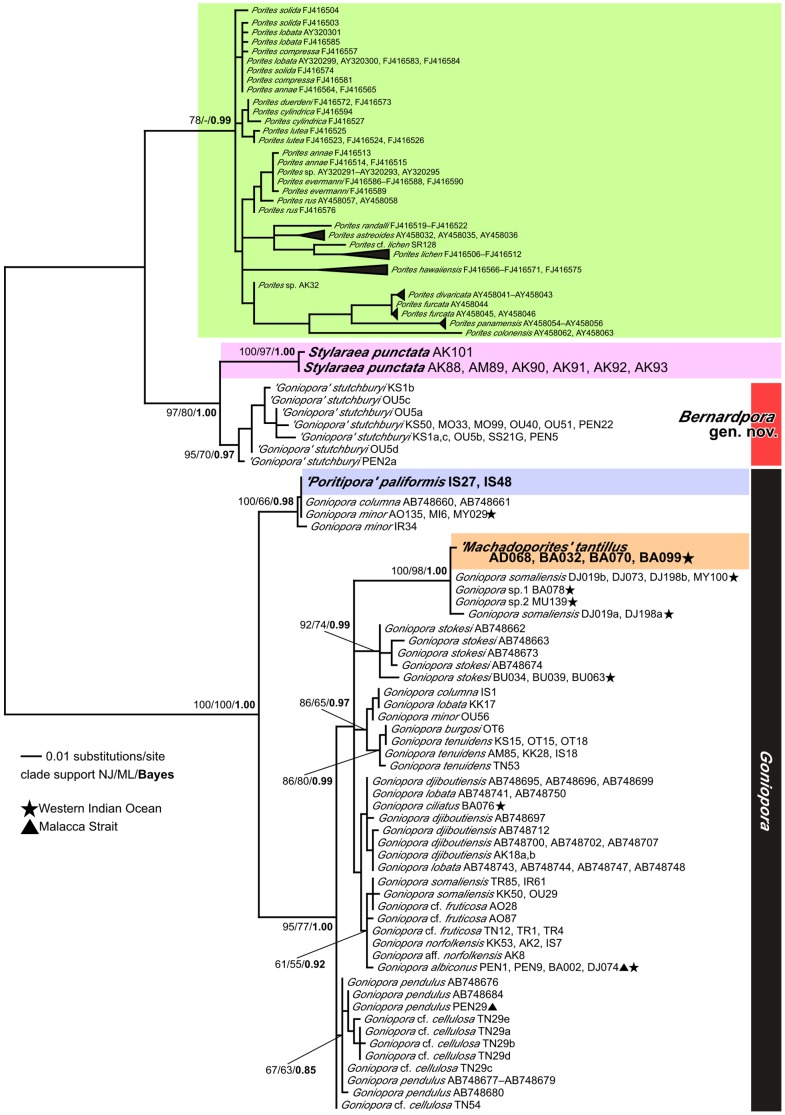
Molecular phylogenetic relationships of genera of the Poritidae except of *Alveopora* based on ITS sequences. Letter (a, b, c, d) after sample code indicates that different alleles were obtained from a single coral sample by cloning. Numbers on/below main branches show bootstrap values (>50%) in ML and NJ analyses, and Bayesian posterior probability (>0.8). Stars show specimens collected from western Indian Ocean, and triangles show ones collected from Malacca Strait. Sample codes or accession numbers are shown after species names (see Table 1). Green in color for *Porites*, purple for *Stylaraea*, blue for ‘*Poritipora*’, orange for ‘*Machadoporites*’, red for *Bernardpora,* and black for *Goniopora*.

The phylogenetic relationships among *Porites*, *Goniopora*, *Stylaraea*, *Poritipora*, and *Machadoporites* were inferred using ITS (Fig. 5). The 68 *Porites* sequences from Forsman et al. [9] and 26 *Goniopora* sequences from Kitano et al. [13] were also added for this analysis (see Table S3). A total of 347 positions were used (108 polymorphic sites with 89 informative sites). This ITS tree also showed similar topology to the COI tree as described above. In particular, *Stylaraea punctata* and *G. stutchburyi* are sister taxa. *Poritipora paliformis* formed a clade with *G. minor* and *G. columna*. One specimen of *G. minor* in the *Poritipora* clade is from the western Indian Ocean and others are from Japan. *Machadoporites tantillus* formed a clade with *G. somaliensis* and another 3 species (*G*. cf. *somaliensis*, *G*. sp.1, and *G*. sp.2), all of which were collected from the western Indian Ocean. Other western Indian Ocean specimens (*G. albiconus*, *G. ciliatus*) and Malacca Strait specimens (*G. albiconus*, *G. pendulus*) were included in a major clade of *Goniopora* spp. Meanwhile, species relationships of *Goniopora* were less resolved because *Porites* and *Goniopora* have many indels in their rDNA sequences and phylogenetic information sites were largely excluded.

The tree using combined data of COI and ITS showed a similar topology to the one for ITS (Fig. S1).

## Discussion

### Phylogenetic relationships of *Goniopora*, *Stylaraea*, and *Porites*

Our molecular data showed that *Goniopora* can be divided into 2 distinct groups (Figs. 4, 5). One contains most *Goniopora* species together with *Machadoporites* and *Poritipora*, whilst the other is monospecific for *G. stutchburyi*. Moreover, *G. stutchburyi* is monophyletic with *Stylaraea punctata* and these 2 species form a sister group to all *Porites*. *Stylaraea punctata* has very unique morphological characters among the zooxanthellate scleractinian corals, such as the smallest (less 1 cm) mature colony size with only 5 or 6 corallites [25 and our observation of the release of zooxanthellate planulae from samples AK92 (maximum diameter 7.15 mm) and AK93 (4.00 mm)], and 12 septa ( = 12 tentacles) without a specific septal pattern such as in *Porites* or *Goniopora*. Moreover, the calices of this species are devoid of pali. Thus, these features allow to distinguish *Stylaraea* from the other genera in this family. Although *Alveopora* can bear resemblance to *Stylaraea,* the two genera can be distinguished based on the columella (not present or very weakly developed in *Alveopora* and star-like columella in *Stylaraea*) and the elongation of polyps (long polyps for *Alveopora* and no elongation for *Stylaraea*). *Goniopora stutchburyi* also has its own specific characters, such as coralla that appear to have a smooth surface, dense septa and a columella composed of a star-shaped central rod (Figs. 2G, H). Thus, *G. stutchburyi* is easily distinguishable from all other congeneric species. Although the smooth appearance of the skeleton of this species and small corallite size may lead to misidentification as *Porites*, *G. stutchburyi* can be differentiated from *Porites* on the basis of its septal number (24 vs. 12). Thus, morphologically, the only character shared by *S. punctata* and *G. stutchburyi* is a columella composed of a star-shaped central rod (Figs. 2D, H), which is absent in other *Goniopora* species but common in *Porites*. This type of columella could be a synapomorphy in the lineage including *Porites*, *Stylaraea*, and *G. stutchburyi*. Considering that molecular and morphological data agree in distinguishing *G. stutchburyi* from the other *Goniopora* species and *S. punctata*, we establish *Bernardpora* Kitano & Fukami gen. nov. (urn:lsid:zoobank.org:act:9C2FE523-A491-45AE-BC22-B528CA68C040, also see below for details) and move *G. stutchburyi* into it. Comparison of diagnostic morphological characters between genera is summarized in Table 2.

Bernard [26] proposed that the septal formula of *Porites* derives from that of *Goniopora* by reduction of the third septal cycle, referring to the typical septal pattern of *Goniopora* as the gonioporoid pattern. Veron and Pichon [15] showed that *G. stutchburyi* typically has this septal pattern (Fig. 2G). In this study, we proved that *G. stutchburyi* is a basal species of *Porites*, and our results strongly support Bernard's hypothesis that *Porites* is derived from *Goniopora*-like morphologies. This conclusion is also supported by the fossil record: *Goniopora* extends back to the Cretaceous, but *Porites* only to the Eocene [16]. Thus, the presence of 24 septa would seem to be an ancestral feature in the Poritidae. The fact that taxa in the family Dendrophylliidae, the closest related outgroup of the Poritidae, have more than 24 septa would appear to support this.

According to Veron and Pichon [15] and Veron [1], *Goniopora burgosi* and *G. somaliensis* are morphologically similar to *G. stutchburyi* in corallite size and height, respectively. Furthermore, *G. burgosi* appears to form only 12 septa as a third cycle of septa is reduced or absent in whole colonies, as is typical of *Porites* (*Goniopora* usually forms 24 septa). However, in our genetic analysis, *G. burgosi* and *G. somaliensis* were included in the major *Goniopora* clade, and their phylogenetic position was distant from *Porites* even though they have fewer than 24 septa. The typical gonioporoid pattern was also observed in some colonies of *G. lobata* and *G. djiboutiensis*, but they were not closely related to *G. stutchburyi*. These results suggest that each of these morphological characters alone (corallite size, depth, and septal formula) would not be sufficient to explain the species relationships in this genus.

### Phylogenetic position of *Alveopora*

None of the 8 species of *Alveopora* we analyzed is closely related to *Goniopora*, despite the 2 genera having similar elongating polyps and tentacles. This suggests that the typically elongated polyps in these genera have appeared independently as result of convergent evolution. *Alveopora* is also genetically distant from *Porites*. Both genera have 12 tentacles (12 septa), but our genetic data indicate that this character is not synapomorphic, which is also supported morphologically by the difference in septal plan in both genera (spin-like for *Alveopora* and fused for *Porites*). Bernard [26] asserted that *Alveopora* did not belong to the Poritidae, and Veron and Pichon [15] also cast doubt on this classification. Along with our genetic data, the morphological differences between *Alveopora* and other genera in the Poritidae, which have previously been noted [15], support the hypothesis that *Alveopora* does not belong in the Poritidae. Wallace [21] also noted that *Alveopora* has the Acroporidae characteristic of synapticulothecate skeleton but does not have coenosteum. As reported by previous studies, the phylogenetic position of *Alveopora* is close to the Acroporidae [18], [19], [31], [62]. The ITS of *Alveopora* is also very divergent, a characteristic shared by acroporids, but not observed in other families [62]. However, the phylogenetic position of *Alveopora* is still unclear because different topologies (forming a sister group with *Astreopora* or outside of *Astreopora*) were also obtained in COI (Fig. 4) and rDNA [62] trees. Our preliminary analyses of rDNA also showed that *Alveopora* was positioned outside of the *Astreopora* (data not shown). A complete evaluation of the phylogenetic position of *Alveopora* is outside the scope of this study. To perform such an evaluation, morphological comparison among all genera of Acroporidae would be necessary (Wallace et al., in preparation).

### Phylogenetic relationships of the monospecific genera *Stylaraea*, *Machadoporites*, and *Poritipora*

*Poritipora* and *Machadoporites* are found within the *Goniopora* lineage in all molecular phylogenetic trees. This is supported by morphology. *Machadoporites* differs from *Goniopora* by having fewer septa (fewer than 24) and smaller calices (<1.7 mm). However, some *Goniopora* species can have superficially similar characters. For example, *G. burgosi* has typically 12–15 septa, as shown in the original description ([51], Fig. 3G). A similar pattern is also observed in *G. pendulus* (Fig. 3H). Moreover, the *G. minor* calices were described as 1.5–2 mm in size in the original description [50]. Thus, characters such as “fewer than 24 septa,” and “small size calices” are not enough to separate *Machadoporites* from *Goniopora*. In addition, *M. tantillus* forms a clade with *G. somaliensis* and other *Goniopora* species from the western Indian Ocean.

Similar to *Goniopora*, *Poritipora* has 24 septa, but the 2 genera can be distinguished by the difference in the number of septal cycles: 2 in *P. paliformis* and 3 in *Goniopora*. However, for several *Goniopora* species, primary and secondary cycles of septa are equal or subequal, such as in the case of *G*. *minor* (Fig. 3D). Therefore, the character “two cycles of septa” is not enough to separate *Poritipora* from *Goniopora*. In addition, *P. paliformis* forms a clade with *G. minor* and *G. columna*.

On the one hand, *Machadoporites* and *Poritipora* are considered junior synonyms of *Goniopora* and their taxonomy is hence revised hereafter.

On the other hand, the type material of *P. paliformis* (Fig. 3C) and our samples (Fig. 3B) look similar to the type material of *G. minor* (Fig. 3D) shown in Crossland [50]. *Goniopora minor* has a similar size of corallites, 12 equally sized septa for the primary and secondary cycles, small or absent septa in tertiaries, and 4–6 pali. The development of the columella was described as “large,” but it is composed only of joined septa, which is the same pattern as that of *Poritipora*. Considering that most *G. minor* examined in this study (one colony of *G. minor* was genetically separated; Figs. 4 and 5) formed a clade with *P. paliformis* with little genetic difference, *P. paliformis* may be a morphological variant of *G. minor*.

### Regional differences

Our specimens were collected mostly from Japanese waters, but some of them including *Machadoporites* were collected from the western Indian Ocean and the Malacca Strait. Although several species from these regions, such as *G. albiconus* and *G. ciliatus*, were included in the clade with specimens collected from Japanese waters, 4 specimens, including *G. somaliensis* from the western Indian Ocean, formed their own clade, whereas *G. somaliensis* from Japanese waters was distant from the western Indian Ocean clade and included in a major clade of *Goniopora* spp. This suggests that morphological convergence may have occurred between the western Pacific and the western Indian Ocean populations of these species. Recently, Arrigoni et al. [75] showed that numerous cases of intraspecific divergence between Indian Ocean and Pacific Ocean populations were present in the families Merulinidae and Lobophylliidae. Moreover, Keshavmurthy et al. [76] reported that a widely distributed species, *Stylophora pistillata*, comprises 4 divergent clades corresponding to different regions, such as the western Pacific and the Red Sea, suggesting that their clades are divergent at the species level. A similar observation on divergence was made with regard to the octocoral family Melithaeidae [77], indicating that species did not cluster according to their present morphological classification but instead clustered according to a biogeographical pattern such as the Indo-Pacific, Red Sea and Indian Ocean. Thus far, many studies of scleractinian corals have focused on higher level taxonomy and are based on material sampled from one or a few nearby Indo-Pacific regions. However, species-level analyses among regions are the next necessary step in the ongoing revolution in scleractinian taxonomy.

### Taxonomic account

Below we propose the description of the new genus *Bernardpora* gen. nov. and the revised diagnosis of *Goniopora*, based on the original descriptions and subsequent information resulting from this study. See Table 1 for the museum abbreviations.

Family Poritidae Gray, 1847

Type genus: *Porites* Link, 1807

Diagnosis [1], [16], [60]: Massive, laminar or ramose colonies; corallites vary in size but usually small and mostly compacted closely without coenosteum, with one or two synapticular rings. Walls and septa are porous. Septa usually 12 to 24. Septa formed by 3 to 8 nearly vertical trabeculae, and innermost trabeculae of certain septa differentiated as pali.

Remarks: There are four extant genera in Family Poritidae, *Porites*, *Goniopora*, *Stylaraea* and *Bernardpora* gen. nov. All are zooxanthellate corals. *Porites* is the only genus distributed throughout the tropics. Others are Indo-Pacific. Based on our results we confirm that the genus *Alveopora* does not belong to the same lineage as the family Poritidae. Although a full evaluation of the position of *Alveopora* is not completed yet, it is certain that *Alveopora* is closely related to other genera in the family Acroporidae ([20], this study).

Genus *Porites* Link, 1807

Type species: *Porites polymorphus* Link, 1807: 163 ( =  *Madrepora porites* Pallas, 1766:324–326, Neotype: MHNNP Lamarck Collection No. 150 (figured in Jameson & Cairns, 2012, figs 4D, 5). This specimen is also the holotype of *Porites clavaria* Lamarck, 1816 [78], [80])

Generic synonymy [2], [16], [78]

- *Neoporites* Duchassaing & Michelotti, 1864: 97. Type species is not fixed.

*- Cosmoporites* Duchassaing & Michelotti, 1864: 99. Type species: *Cosmoporites laevigata* Duchassaing & Michelotti, 1864: 99. Holotype: unknown (figured in Duchassaing & Michelotti, 1864: 99, pl. x, figs. 12, 16. Bernard [79] described ‘the type specimen was not found by Count Peracca in the Turin Museum’.)

- *Synaraea* Verrill, 1864: 42. Type species: *Porites erosa* Dana 1846: 565–566, pl. 55, fig. 8. Holotype: USNM 668

- *Napopora* Quelch, 1884:296. Type species: *Napopora irregularis* Quelch, 1884: 296–297. Holotype: NHMUK 86.12.9.302.

Diagnosis [1], [16], [78]: Colonies massive, ramose, laminar, or encrusting. Corallites are small, immersed, circular or polygonal. Calice diameter 0.5–2.2 mm. Septa are 12 in number, composed of 1 to 4 trabeculae. The typical formula of septal arrangement in this genus, with some of its variations, is seen. Pali are present, variable development in different species, usually 4–8 in number. Mural trabeculae always present. Columella trabeculae usually present with star-shaped granules. The wall is really simple, but the incipient synapticulae, seen starting from the sides of septal granules, may become complete and form an inner synapticular wall.

Remarks: Distribution: Indo-Pacific and Atlantic [1]. Species number: 73[1], [15]

Genus *Goniopora* de Blainville, 1830

Type species: *Goniopora pedunculata* Quoy & Gaimard, 1833:218–220, pl. 16, Figs. 9–11. The type specimen appears to be lost [15].

Generic synonymy [2], [16], [26]

- *Rhodaraea* Mile Edwards & Haime, 1849: 259. Type species: *Astraea calicularis*, Lamarck 1816: 266. Holotype: unknown.

- *Tichopora* Quelch, 1886:188. Type species: *Tichopora tenella* Quelch, 1886: 189, pl. 11, figs. 1, 1a. Type specimens: NHMUK 86.12.9.342.

*- Poritipora* Veron, 2000:347. Type species: *Poritipora paliformis* Veron, 2000: 347. lectotype: MTQ G55857

- *Calathiscus* Claereboudt & Al-Amri, 2004. Type species: *Calathiscus tantillus* Claereboudt & Al-Amri, 2004 (This species is also type species of the genus *Machadoporites*). Holotype: SQU040001.

- *Machadoporites* Nemésio, 2005. Type speices: *Calathiscus tantillus* Claereboudt & Al-Amri, 2004.

Revised diagnosis [1,16,26,this study]: Massive, columnar or ramose, rarely encrusting colonies. Corallites are circular or polygonal. Calice diameter 1–10 mm. Septa 24 in two or three cycles, or between 24 and 12 in two or three cycles, composed of 4 to 8 trabeculae. Pali and columella may develop. Columellae are composed of anastomosed septal dentations or arranged synapticula and fused inner ends of septa. Wall structure is synapticulothecal. Polyps usually elongate during the day (note that *G. paliformis* does not elongate polyps during the day).

Remarks: *Poritipora* and *Machadoporites* are considered as junior synonyms of *Goniopora*. Distribution: Indo-Pacific [1]. Species number: 33 [1,15,this study].

Genus *Stylaraea* Milne Edwards & Haime, 1851

Type species: *Madrepora punctata* Linnaeus, 1758:795. The specimen ZMB #956 may be Syntype [78] (examined).

Diagnosis: *Stylaraea* is a monospecific genus with only known species, *S. punctata*. Therefore, the characters of this genus are those of *S. punctata*. Colonies are tiny (usually less 10 mm in size) and from “cushion-shaped crusts”[46]. Calices are concavate and around 1 mm diameters. Septal number is 12 (“2 cycles of 6 each” [15]) without specific septal pattern. Septa are composed of rows of star-shaped granules. Primary septa may reach to collumellae. Columella is composed of a star-shaped central rod such as *Porites* or *Bernaldopora*. Wall structure is synapticulothecal.

Remarks: Distribution: Indo-Pacific [1]. Species number: 1

Genus *Bernardpora* Kitano & Fukami gen. nov.

urn:lsid:zoobank.org:act:9C2FE523-A491-45AE-BC22-B528CA68C040

Type species: *Goniopora stutchburyi* Wells, 1955: 11, pl. 1, figs 1–2; Holotype: MTQ G2931 (examined)

Diagnosis: *Bernardpora* is a monospecific genus with only known species, *B. stutchburyi*. Therefore, the characters of this genus are those of *B. stutchburyi*. Encrusting or sub-massive colonies. Corallites round to polygonal and very shallow with smooth appearance. Calices are around 2 mm diameters. Septal number is mostly 24 with clear gonioporoid pattern shown in Bernard [26]. Septa are dense with up to seven multiple-spine-shaped septal teeth. Septal teeth closest to collumellae are indistinguishable from pali. Primary and secondary septa nearly reach to collumellae. Columella is composed of a star-shaped central rod such as *Stylaraea* and *Porites*. Wall structure is synapticulothecal. Polyps elongate but very short during the day.

Remarks: Distribution: Indo-Pacific [1]. Species number: 1

Etymology: The generic name is in honor of the coral scientist Henry M. Bernard.

## Supporting Information

Figure S1 **Molecular phylogenetic relationships of genera of the Poritidae except of** ***Alveopora*** **based on combined COI+ITS sequences.** Numbers on/below main branches show bootstrap values (>50%) in ML and NJ analyses, and Bayesian posterior probability (>0.8). Stars show specimens collected from western Indian Ocean, and triangles show ones collected from Malacca Strait. Sample codes or accession numbers are shown after species names (see Table 1, Table S3). Grey in color for *Alveopora*, green for *Porites*, purple for *Stylaraea*, blue for ‘*Poritipora*’, and orange for ‘*Machadoporites*’. *Goniopora* is shown by bars in black. *Bernardpora* is shown by bar in red.
(TIF)

Dataset S1 **Nexus data file of the COI sequence alignments used for the analyses.**
(NXS)

Dataset S2 **Nexus data file of the ITS sequence alignments used for the analyses.**
(NXS)

Table S1 Summary of the diagnostic morphological characters for species identification of the genus *Goniopora*. Original descriptions are shown in bold.
(XLS)

Table S2 Summary of the diagnostic morphological characters for species identification of the genus *Alveopora*. Original descriptions are shown in bold.
(XLS)

Table S3 List of poritid samples and accession numbers for COI and ITS, referred from previous study.
(DOCX)
