# Supplementary figures and images for: A Phylogeny of the Family Poritidae (Cnidaria, Scleractinia) Based on Molecular and Morphological Analyses
Source: PLoS One. 2014 May 28;9(5):e98406. doi: 10.1371/journal.pone.0098406 (PMC4037213; doi:10.1371/journal.pone.0098406)

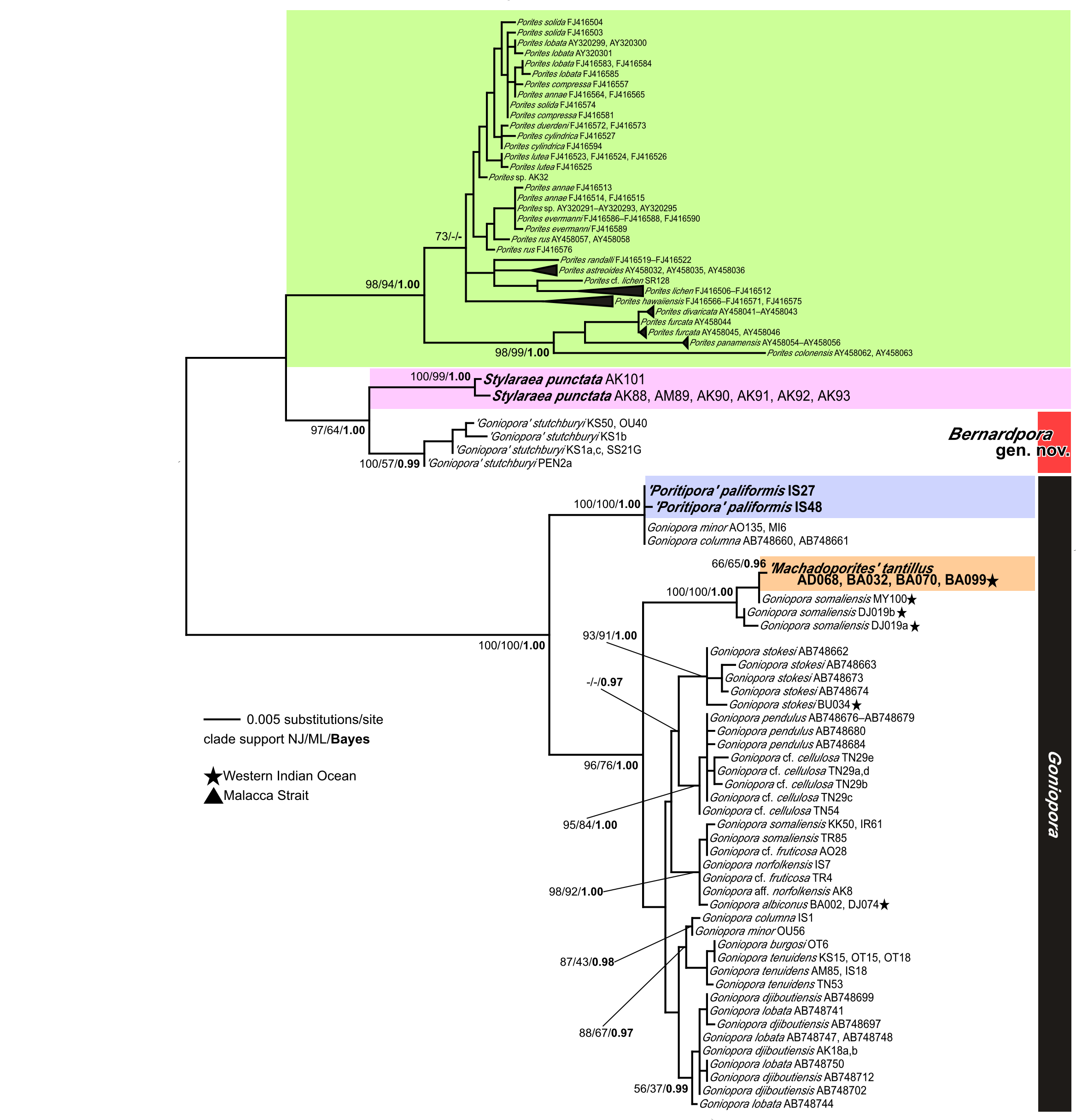

Supplement: Figure S1 — Molecular phylogenetic relationships of genera of the Poritidae except of Alveopora based on combined COI+ITS sequences. Numbers on/below main branches show bootstrap values (>50%) in ML and NJ analyses, and Bayesian posterior probability (>0.8). Stars show specimens collected from western Indian Ocean, and triangles show ones collected from Malacca Strait. Sample codes or accession numbers are shown after species names (see Table 1, Table S3). Grey in color for Alveopora, green for Porites, purple for Stylaraea, blue for ‘Poritipora’, and orange for ‘Machadoporites’. Goniopora is shown by bars in black. Bernardpora is shown by bar in red. (TIF) [file pone.0098406.s001.tif]
