# Supplementary material for: A Phylogeny of the Family Poritidae (Cnidaria, Scleractinia) Based on Molecular and Morphological Analyses
Source: PLoS One. 2014 May 28;9(5):e98406. doi: 10.1371/journal.pone.0098406 (PMC4037213; doi:10.1371/journal.pone.0098406)
Supplement: Table S3 — List of poritid samples and accession numbers for COI and ITS, referred from previous study. (DOCX) [file pone.0098406.s006.docx]

Table S3. List of poritid samples and accession numbers for COI and ITS, referred from previous study.

| family | genus | species | sample No. | COI | ITS | reference |
| --- | --- | --- | --- | --- | --- | --- |
| Poritidae | *Goniopora* | *columna* | IS3 | - | AB748660 | [13] |
| Poritidae | *Goniopora* | *columna* | IS4 | AB748754 | AB748661 | [13] |
| Poritidae | *Goniopora* | *djiboutiensis* | AM74 | AB748776 | AB748699 | [13] |
| Poritidae | *Goniopora* | *djiboutiensis* | IR67 | AB748780 | AB748712 | [13] |
| Poritidae | *Goniopora* | *djiboutiensis* | KK16 | - | AB748700 | [13] |
| Poritidae | *Goniopora* | *djiboutiensis* | KS9 | - | AB748695 | [13] |
| Poritidae | *Goniopora* | *djiboutiensis* | OT30 | AB748775 | AB748697 | [13] |
| Poritidae | *Goniopora* | *djiboutiensis* | OU21 | AB748778 | AB748705 | [13] |
| Poritidae | *Goniopora* | *djiboutiensis* | OU33 | - | AB748707 | [13] |
| Poritidae | *Goniopora* | *djiboutiensis* | SR62 | - | AB748696 | [13] |
| Poritidae | *Goniopora* | *djiboutiensis* | SS35 | AB748777 | AB748702 | [13] |
| Poritidae | *Goniopora* | *lobata* | IR42 | AB748791 | AB748750 | [13] |
| Poritidae | *Goniopora* | *lobata* | KS28 | AB748788 | AB748741 | [13] |
| Poritidae | *Goniopora* | *lobata* | MO36 | AB748790 | AB748747-8 | [13] |
| Poritidae | *Goniopora* | *lobata* | SR44 | - | AB748743 | [13] |
| Poritidae | *Goniopora* | *lobata* | YO1 | AB748789 | AB748744 | [13] |
| Poritidae | *Goniopora* | *pendulus* | OT14 | AB748764 | AB748676-80 | [13] |
| Poritidae | *Goniopora* | *pendulus* | TN11 | AB748767 | AB748684 | [13] |
| Poritidae | *Goniopora* | *stokesi* | NK1 | AB748755 | AB748662 | [13] |
| Poritidae | *Goniopora* | *stokesi* | OU12 | AB748756 | AB748663 | [13] |
| Poritidae | *Goniopora* | *stokesi* | IR27 | AB748762 | AB748673-4 | [13] |
| Poritidae | *Porites* | *annae* | as31 | FJ423964 | FJ416513-5 | [9] |
| Poritidae | *Porites* | *annae* | hm19 | FJ423975 | FJ416564-5 | [9] |
| Poritidae | *Porites* | *astreoides* | p2 | FJ423989 | AY458032 | [9] |
| Poritidae | *Porites* | *astreoides* | br6 | FJ423961 | AY458035-6 | [9] |
| Poritidae | *Porites* | *colonensis* | colP3 | FJ423972 | AY458062-3 | [9] |
| Poritidae | *Porites* | *compressa* | cit | FJ423970 | FJ416557 | [9] |
| Poritidae | *Porites* | *compressa* | l2 | FJ423971 | FJ416581 | [9] |
| Poritidae | *Porites* | *cylindrica* | wa4 | FJ423996 | FJ416594 | [9] |
| Poritidae | *Porites* | *cylindrica* | as43 | FJ423968 | FJ416527 | [9] |
| Poritidae | *Porites* | *divaricata* | pb4 | FJ423969 | AY458041-3 | [9] |
| Poritidae | *Porites* | *duerdeni* | hm29 | FJ423977 | FJ416573 | [9] |
| Poritidae | *Porites* | *duerdeni* | hm28 | FJ423976 | FJ416572 | [9] |
| Poritidae | *Porites* | *evermanni* | l6 | FJ423985 | FJ416587-90 | [9] |
| Poritidae | *Porites* | *evermanni* | l5 | FJ423984 | FJ416586 | [9] |
| Poritidae | *Porites* | *furcata* | p1 | FJ423988 | AY458044-6 | [9] |
| Poritidae | *Porites* | *hawaiiensis* | hm20 | FJ423981 | FJ416566-71 | [9] |
| Poritidae | *Porites* | *hawaiiensis* | hm54 | FJ423979 | FJ416575 | [9] |
| Poritidae | *Porites* | *lichen* | as30 | FJ423987 | FJ416508-12 | [9] |
| Poritidae | *Porites* | *lichen* | as29 | FJ423963 | FJ416506−7 | [9] |
| Poritidae | *Porites* | *lobata* | e47 | FJ423973 | Y320299-301 | [9] |
| Poritidae | *Porites* | *lobata* | l4 | FJ423983 | FJ416583-5 | [9] |
| Poritidae | *Porites* | *lutea* | as36 | FJ423967 | FJ416523-6 | [9] |
| Poritidae | *Porites* | *panamensis* | pan75 | FJ423990 | AY458054-6 | [9] |
| Poritidae | *Porites* | *rus* | hm55 | FJ423980 | FJ416576 | [9] |
| Poritidae | *Porites* | *rus* | rus1 | FJ423993 | AY458057-8 | [9] |
| Poritidae | *Porites* | *solida* | hm35 | FJ423978 | FJ416574 | [9] |
| Poritidae | *Porites* | *solida* | as18 | FJ423962 | FJ416503−4 | [9] |
| Poritidae | *Porites* | sp. | pp47 | FJ423992 | AY320295 | [9] |
| Poritidae | *Porites* | sp. | pp19 | FJ423991 | AY320291-3 | [9] |
| Poritidae | *Porites* | *randalli* | as35 | FJ423966 | FJ416519-22 | [9] |
